# Circulating S100B and Adiponectin in Children Who Underwent Open Heart Surgery and Cardiopulmonary Bypass

**DOI:** 10.1155/2015/402642

**Published:** 2015-08-31

**Authors:** Alessandro Varrica, Angela Satriano, Alessandro Frigiola, Alessandro Giamberti, Guido Tettamanti, Luigi Anastasia, Erika Conforti, Antonio D. W. Gavilanes, Luc J. Zimmermann, Hans J. S. Vles, Giovanni Li Volti, Diego Gazzolo

**Affiliations:** ^1^Department of Pediatric Cardiac Surgery IRCCS San Donato Milanese Hospital, Via Morandi 30, 20097 San Donato Milanese, Italy; ^2^Department of Pediatrics, Neonatology and Child Neurology, Maastricht University, P. Debyelaan 25, 5800 Maastricht, Netherlands; ^3^Department of Drug Sciences, Section of Biochemistry, University of Catania, Viale A. Doria 6, 95125 Catania, Italy; ^4^Department of Maternal Fetal and Neonatal Medicine, C. Arrigo Children's Hospital, Spalto Marengo 46, 15100 Alessandria, Italy

## Abstract

*Background*. S100B protein, previously proposed as a consolidated marker of brain damage in congenital heart disease (CHD) newborns who underwent cardiac surgery and cardiopulmonary bypass (CPB), has been progressively abandoned due to S100B CNS extra-source such as adipose tissue. The present study investigated CHD newborns, if adipose tissue contributes significantly to S100B serum levels. *Methods*. We conducted a prospective study in 26 CHD infants, without preexisting neurological disorders, who underwent cardiac surgery and CPB in whom blood samples for S100B and adiponectin (ADN) measurement were drawn at five perioperative time-points. *Results*. S100B showed a significant increase from hospital admission up to 24 h after procedure reaching its maximum peak (*P* < 0.01) during CPB and at the end of the surgical procedure. Moreover, ADN showed a flat pattern and no significant differences (*P* > 0.05) have been found all along perioperative monitoring. ADN/S100B ratio pattern was identical to S100B alone with the higher peak at the end of CPB and remained higher up to 24 h from surgery. *Conclusions*. The present study provides evidence that, in CHD infants, S100B protein is not affected by an extra-source adipose tissue release as suggested by no changes in circulating ADN concentrations.

## 1. Introduction

Advances in cardiothoracic surgical and anesthetic techniques, including cardiopulmonary bypass (CPB), have substantially decreased mortality, expanding the horizon to address functional neurologic and cardiac outcomes in long-term survivors [1]. Acute neurocardiac morbidities in congenital heart disease (CHD) infants are well described and interest in the functional status of survivors now stretches beyond the newborn period to childhood, adolescence, and adulthood [2]. Newborn heart surgery represents a period of planned and deliberate hypoxia-ischemia (HI) injury, which is the price to pay in the treatment or palliation of CHD. To date, the possibility of detecting infants at risk for mortality and morbidity is limited since clinical, laboratory, and standard monitoring procedures may be silent or unreliable [3]. Thus, a practical and sensitive marker able to offer physicians a useful tool for clinical is therefore eagerly awaited.

In the last decade a brain constituent, namely, S100B protein, has been proposed as a well-established marker of brain damage and death [4–10], since elevated S100B concentrations in different biological fluids have been found in adults, infants, and fetuses at risk for brain damage [4–15]. S100B is an acidic calcium-modulated protein of low molecular weight, first identified by Moore as a protein fraction detectable in the central nervous system (CNS) in glial and Schwann cells and in specific neuronal subpopulations [16]. With regard to CHD infants who underwent cardiac surgery and CPB, S100B has been shown to be increased in the perioperative period [17, 18], to correlate with different CPB phases [19] and with increased cerebrovascular resistance and brain damage [20]. However, protein's assessment in CHD infants for CPB monitoring has been progressively abandoned on the basis of S100B extra CNS site of concentrations including adipose tissue [21]. The issue is still controversial and matter of debate. From one side, a contamination originating by mediastinal tissues on S100B releasing into systemic circulation has been suggested [22–25]. From the other side, it has been shown that extracranial sources of S100B do not affect serum levels and protein's diagnostic value in neurological diseases in intact subjects [26]. In this setting, data in nonintact patients such as without traumatic brain or bodily injury from accident or surgery are still lacking.

Therefore, the objective of this current study was to determine, in CHD newborns, if adipose tissue sources contribute significantly to serum levels of S100B by means of the longitudinal measurement of adiponectin (ADN), the most abundant adipose-derived protein in humans [27] and S100B at different perioperative time-points.

## 2. Materials and Methods

### 2.1. Patients

From March 2010 to September 2011, we conducted an observational study in which 26 infants (15 males and 11 females) from 0 to 9 months of age (mean 30.4 months), without preexisting neurological disorders or other comorbidities, admitted to our referral centers for the correction of congenital heart defects (Table 1). Exclusion criteria included need for inotropic support or mechanical ventilation prior to surgery, recent cardiac arrest, and weight of less than 2 kg.

Informed consent from parents was obtained before patient inclusion in the study, which was approved by the local human-investigation committee.

Blood samples were drawn at five predetermined time-points in the preoperative period as follows: before the surgical procedure (time 0,* T*0); during the surgical procedure before CPB (time 1,* T*1); at the end of CPB (time 2,* T*2); at the end of the surgical procedure (time 3,* T*3); 24 h after the surgical procedure (time 4,* T*4). At these time-points ADN levels and ADN/S100B ratio were measured. Clinical parameters (peripheral temperature, nasopharyngeal temperature, pump flow rate, mean blood pressure, and arterial pH) were recorded at all sample times for the purpose of monitoring the general pattern of the surgical procedure.

#### 2.1.1. Anesthetic Technique

After premedication with midazolam 0.5 mg/Kg bw (rectal/intramuscular), induction was achieved with oxygen and 3% sevofluorane administered via mask (single breath induction), followed by intravenous sufentanil 1 (g/Kg bw) and vecuronium (0.15 mg/Kg bw). Maintenance was achieved with 3% sevofluorane (except during CPB) and with additional doses of sufentanil (0.5 g/Kg bw) and vecuronium (0.1 mg/Kg bw) every 30–40 min. During CPB, in the absence of sevofluorane, additional midazolam at 0.2 mg/Kg bw dosage was given. Sufentanil infusion at 0.25 g/Kg bw was continued in the intensive care unit for sedation.

#### 2.1.2. Cardiopulmonary Bypass Management

CPB was established after systemic heparinization (3 mg/Kg bw) by standard single stage aortic and bicaval cannulation and was maintained via nonpulsatile pump flow with a membrane oxygenator (Dideco Laboratories, Modena, Italy). Flow velocity was kept at 120–150 mL/Kg bw and mean arterial blood pressure at 45 mmHg; hypothermia was attained by core and surface cooling. Mean CPB duration time was 90 ± 43 min; mean rewarming time was 15 ± 8 min (mean ± SD), calculated from the final temperature during hypothermic circulatory arrest to 36.5°C. The minimum temperature reached was 27.2°C. The pump priming solution was composed of electrolyte solutions (Normosol-R 250 to 650 mL, Abbott Hospital Products, Abbott Park, IL, USA or Plasma-Lyte A, Travenol Laboratories, Inc., Deerfield, IL, USA), albumin (25%), heparin 1000 to 5000 units in the total solution, sodium bicarbonate (25–30 mEq/L), and packed red blood cells or fresh frozen plasma. A standard circuit prime total volume was used, according to body-weight varying from 400 mL (bw < 4.5 Kg) to 600 mL (bw > 4.5 Kg and bw < 7.5 kg) and to 700 mL (bw > 7.7 kg). Packed red blood cells (200 to 500 mL) were transfused as necessary to maintain a hematocrit level above 30% during CPB [26]. Protamine (1 mg for each mg of heparin) was administered at the end of CPB.

The *α*-stat regimen was used, and the PaCO_2_ was maintained between 35 and 40 mmHg, without mathematical correction for the effects of the temperature, by varying the membrane oxygenator gas flow.

#### 2.1.3. Adiponectin Measurement

Serum ADN concentrations were determined by an enzyme-linked immunosorbent assay (Human Adiponectin ELISA, EZHADP-61 K; Linco Research). Sensitivity limit for this assay is 0.78 ng/mL for Human Adiponectin (20 *μ*L sample size). The appropriate range of the assay is 1.56 to 200 ng/mL Human Adiponectin (20 *μ*L sample size). The results were evaluated according to ng/mL.

#### 2.1.4. S100B Measurement

Samples for S100B measurements at the seven monitoring time-points were drawn from a catheter inserted in the jugular vein. Heparin-treated blood samples were immediately centrifuged at 900 g for 10 min and the supernatants were stored at −70°C before measurement. The S100B protein concentration was measured in all samples using a commercially available two-site immunoradiometric assay kit (Sangtec 100; AD Sangtec Medical, Bromma, Sweden) specific to the *β*-subunit of the protein, which is known to be present mostly (80–96%) in the human brain [28]. Each measurement was performed in duplicate and the mean values are reported. The limit of sensitivity of the assay was 0.02 *μ*g/L. The precision (CV) was <10%.

#### 2.1.5. Neurological Follow-Up

Neurological development was assessed by physical examination, preoperatively and on the 7th postoperative day, based on Amiel-Tison's criteria [29]. In particular, resistance against passive movements, visual pursuit, reaching and grasping, and responses to visual and acoustic stimuli were tested by the same examiner, who did not know of the subjects' presurgical condition.

### 2.2. Statistical Analysis

ADN and S100B plasma concentrations are expressed as median and 5–95% coefficient intervals (CI). Comparisons at the different monitoring time-points were analyzed by Kruskal-Wallis one-way ANOVA. Linear regression analysis was used for correlation between ADN and S100B and various parameters (CPB, cooling, and rewarming duration; body core temperature; arterial blood pH, arterial oxygen and carbon dioxide partial pressures, and base excess; and mean arterial blood pressure and heart rate). Statistical significance was set at *P* < 0.05.

## 3. Results

In Table 1 patients' characteristics are reported. Clinical, laboratory, and standard monitoring parameters recorded at the predetermined time-points remained within the reference limits and therefore were not different (*P* > 0.05; for all) in all infants. Intraoperative parameters such as CPB, cross-clamping, cooling, and rewarming durations were within reference ranges and no perioperative complications have been shown. No complications in the postoperative period have been reported and no overt neurological disease was detected at discharge from hospital.

**Figure 1:**
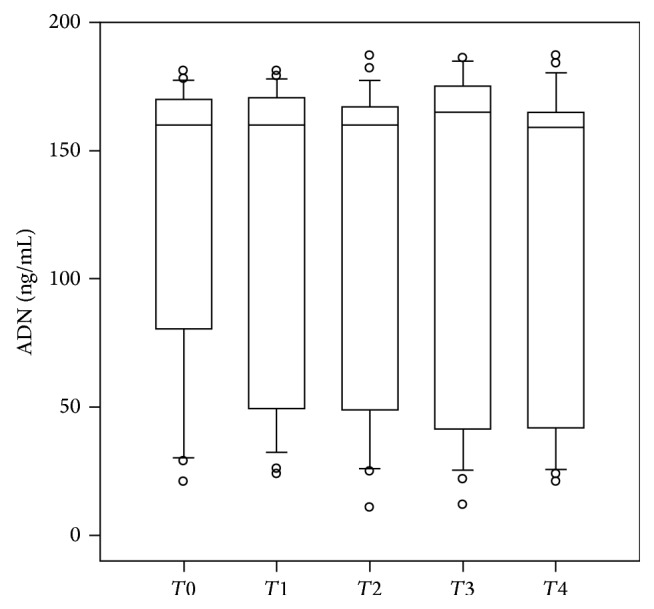
Adiponectin (ADN) (ng/mL) blood concentrations expressed as median and coefficient intervals (CI_5–95%_) at different monitoring time-points (before the surgical procedure (*T*0); during the surgical procedure after sternotomy before CPB (*T*1); at the end CPB (*T*2); at the end of the surgical procedure (*T*3); and at 24 h after the surgical procedure (time 4,* T*4)).

ADN was measurable in all samples collected. ADN pattern at different monitoring time-points showed a flat trend and therefore no significant differences (*P* > 0.05, for all) have been found all along perioperative monitoring up to 24 h from surgery (*T*0–*T*4) (Figure 1).

**Figure 2:**
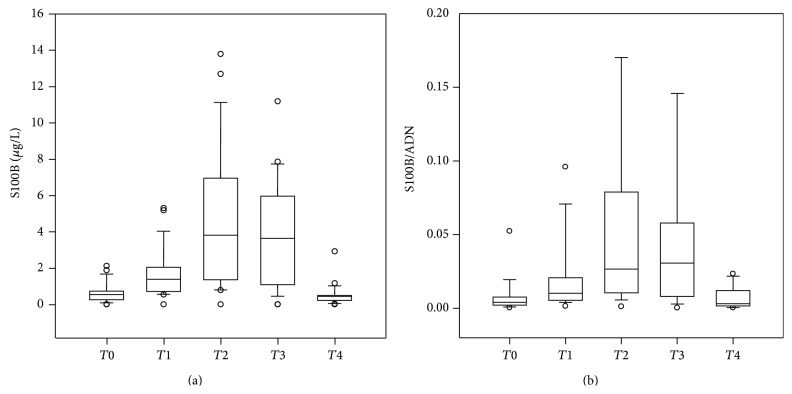
(a) S100B (*μ*g/L) blood concentrations expressed as median and coefficient intervals (CI_5–95%_) at different monitoring time-points (before the surgical procedure (*T*0); during the surgical procedure after sternotomy before CPB (*T*1); at the end CPB (*T*2); at the end of the surgical procedure (*T*3) and at 24 h after the surgical procedure (time 4,* T*4)). ^*^ *P* < 0.01 versus* T*0. (b) S100B (*μ*g/L) and adiponectin (ADN) (ng/mL) blood concentrations expressed as median and coefficient intervals (CI_5–95%_) at different monitoring time-points (before the surgical procedure (*T*0); during the surgical procedure after sternotomy before CPB (*T*1); at the end CPB (*T*2); at the end of the surgical procedure (*T*3); and at 24 h after the surgical procedure (time 4,* T*4)). ^*^ *P* < 0.01 versus* T*0.

S100B was measurable in all samples collected. S100B pattern at different monitoring time-points was characterized by a protein's significant increase (*P* < 0.01, for all), reaching its highest peak at the end of CPB and remaining stable up to 24 h from surgery (Figure 2(a)).

Linear regression analysis showed no significant correlations (*P* > 0.05, for all) between ADN and S100B at all monitoring time-points (*T*0–*T*4) and between ADN and CPB (*r* = 0.08; *P* = 0.73) and cross clamp (*r* = 0.05; *P* = 0.82) durations. Conversely, S100B significantly correlated with CPB (*r* = 0.53; *P* = 0.003) and at cross clamp (*r* = 0.65; *P* < 0.01) durations.

ADN/S100B ratio pattern was characterized by a significant increase (*P* < 0.01) from* T*0 to* T*3 reaching its dip at* T*2 and returning at* T*4 at preoperative levels. No significant correlations (*P* > 0.05, for all) between ADN/S100B ratio and CPB (*r* = 0.12; *P* = 0.56) and cross clamp (*R* = 0.19; *P* = 0.35) duration have been found (Figure 2(b)).

## 4. Discussion

Despite recent advances in cardiac surgery and CPB management, the possibility of detecting infants at risk for neonatal mortality and morbidity is still faraway due to limitations in the standard monitoring procedures currently performed [1, 2]. In this setting, brain biomarkers previously suggested as promising tools disappointed expectations and, to date, a trustable biomarker of brain damage in the perioperative period is still eagerly awaited. This holds for S100B protein, first reported as a useful tool and later on abandoned for brain monitoring of CHD adults and children [18–21]. The explanations are still controversial and debated although the main resides in a contamination by protein' extrasources such as adipose tissue [22–25].

The present study provides evidence that, in CHD infants, S100B protein is not affected by an extrasource adipose tissue release as suggested by no changes in circulating ADN concentrations. Furthermore, the ADN/S100B ratio pattern was superimposable to S100B alone all along the perioperative period.

The finding of ADN trend in the perioperative period is not surprising and fits, in part, previous observations in pediatric patients where decreased ADN levels have been reported [27]. The discrepancies are several and reside in the number, timing, and the length of the monitoring time-points and in the different CPB management (mild versus moderate hypothermia). In this setting, hypothermia is known to activate an exaggerated release of proinflammatory cytokines and of endogenous cortisol that may be responsible of decreased ADN transcription and blood levels [27, 30, 31]. Anyway, further investigations comparing ADN pattern under different CPB management such as mild versus moderate/deep hypothermia are so justified.

The finding of increased S100B levels and flat ADN/S100B ratio enforces the debating issue on the protein'* pros and cons *as brain stress/damage marker in CHD patients. From one hand, the absence of any interference in circulating S100B in the perioperative period is in agreement with previous observations, both in adults and children, reporting no compromise on the diagnostic value of S100B in neurological diseases* in intact subjects* (without traumatic brain or bodily injury from accident or surgery) [26]. These findings are also consistent with the usefulness of the protein in brain monitoring of CHD infants [18–20]. On the other hand, the discrepancy with previous observations warrants further consideration in terms of contamination following invasive procedures during CPB. This refers to CPB standard procedures, known to increase mediastinum release of the protein, as pericardial suction blood re-/autotransfusion, zero-balanced ultra-filtration, and pericardial blood processing with cell-saving devices [22–25, 32–34]. The high S100B levels at the site of reinfusion is* per se* of limited relevance because of the known mediastinum site of concentration of the protein. In fact, once S100B was measured in systemic circulation, after reinfusion procedures, its concentration did not appear to be affected by mediastinum source [35]. The main explanations reside in lowest S100B extrasources' concentrations when compared with the total amount of the protein in the CNS [36]. Although, there are no observations in pediatric and postnatal periods in whom protein distribution in CNS and other tissues can differ or not from adults [37], in the latter (estimated for a 70 Kg man) the absolute amount of S100B in the tissue (calculated in micrograms) showed the highest protein's concentration in brain (538.000 *μ*g: 90.9%) followed by muscles (42.000 *μ*g: 7.1%), adipose tissue (10.500 *μ*g: 1.77%), heart (1.000 *μ*g: 0.2%), and liver (200 *μ*g: 0.03%) [36]. Taken together, the possibility that adipose tissue could constitute a significant source of contamination affecting S100B diagnostic value seems to be fairly remote.

Among different S100B sites of contamination cardiac tissue extrasource deserves further consideration [36]. In particular (i) in rat model of myocardial infarction it has been shown that S100B may play a dual role in cardiomyocytes survival or death (i.e., necrosis and apoptosis) through a RAGE-dependent mechanism, and (ii) S100B, once released from damaged myocytes, with consequent leakage of the protein into the systemic circulation, is approximately 1000-fold less than the amount of protein required to induce apoptosis [38]. Indeed, local high S100B concentration was detected only at the site of myocardial infarction and, finally, (iii) in humans, cardiac tissue contamination on S100B has been justified by a correlation between troponin I and S100B [23] although a hypoxia mediated effect could be reasonably the main explanation of the increase in circulating biomarkers' levels [3, 4]. Altogether, bearing in mind the extremely low protein' concentrations in cardiac tissue the present findings suggest that the possibility of a cardiac tissue extrasource contamination on circulating S100B levels argue against this hypothesis. Conversely, it is reasonable to suppose that the upregulation of S100B protein is a consequence of hypoxia itself, instead of dead cells belonging to the necrosis/apoptosis area. To this regard, it has been demonstrated both in humans and in sheep model that acute hypoxemia is able to induce a significant increase in S100B release within 15 minute form insult in absence of any CNS damage [39]. This is of great relative interest to this contest because, as shown in our series, it is possible to argue that H-I insult occurring during CPB phase may trigger protein's release due to “multiorgan” stress of whom CNS constitutes the majority of the total protein's amount [3, 4, 36]. In this regard, further studies aimed at investigating potential confounding factors such as S100A1 and RAGE and the S100A1B dimer interactions in the cascade of events leading to cell death and apoptosis are still needed.

In conclusion, our results showing, in nonintact patients, that S100B protein is not affected by an extrasource adipose tissue release during the perioperative period open up further studies, in wider populations, aimed at confirming protein's role of early marker of hypoxia and CNS stress/damage in CHD children.

## Figures and Tables

**Table 1:** General characteristics and perioperative data in the infants admitted into the study. Data are given as mean ± SD.

Parameters	
Age (months)	41.6 ± 30.4
Sex (male/female)	15/11
Weight (Kg)	11.5 ± 4.7
Type of surgery	
Great artery transposition (no)	4
Ventricular septal defect (no)	5
Total anomalous pulmonary vein connection (no)	4
Tetralogy of fallot (no)	2
Double outlet right ventricle (no)	3
Complete A-V canal defect (no)	2
Atrial septal defect (no)	6
CPB duration (min) (no)	89.9 ± 43.4
Cross clamp duration (min)	45.1 ± 32.2
Temperature in CPB (°C) median	31.8 ± 3.5
MUF (no)	20

AV: atrioventricular; CPB: cardiopulmonary bypass; and MUF: modified ultrafiltration.

## List of Abbreviations

CPB: Cardiopulmonary bypass
CHD: Congenital heart diseases
CNS: Central nervous system
ADN: Adiponectin
SD: Standard deviation
CI: Coefficient intervals.
